# 

**Published:** 1904-03

**Authors:** 


SUBSCRIPTION $1.00.

PUBLISHED MONTHLY

### ATLANTA JOURNAL-RECORD OF MEDICINE.

Successor to Atlanta Medical and Surgical Journal, Established 1855, and Southern Medical Record, Established 1870.

VOL. V. ATLANTA, GA., MARCH, 1904. No. 12.

